# Non-receptor tyrosine kinase c-Abl downstream of C-type lectin receptors regulates innate antifungal immunity through c-Cbl/MAPK pathway

**DOI:** 10.1128/iai.00365-25

**Published:** 2026-01-26

**Authors:** Shu-Jun Ma, Ke-Fang Xie, Jie-Lin Duan, Xian-Long Wang, Yi-Heng Yang, Ying Wang

**Affiliations:** 1 Department of Dermatology, The First Affiliated Hospital of Naval Military Medical University 12520 , Shanghai, China; 2 Department of Dermatology, The 926th Hospital of PLA 623453 , Kaiyuan, China; 3 Clinical Medicine Scientific and Technical Innovation Center, Shanghai Tenth People's Hospital, School of Medicine, Tongji University 481875, Shanghai, China; 4 School of Pharmacy, China Pharmaceutical University 428683 , Nanjing, China; 5 Department of Respiratory and Critical Care Medicine, Institute of Respiratory Medicine, Dongguan Key Laboratory of Immune Inflammation and Metabolism, The First Dongguan Affiliated Hospital, Guangdong Medical University, Dongguan, China; University of Pennsylvania Perelman School of Medicine, Philadelphia, Pennsylvania, USA

**Keywords:** c-Abl, *C. albicans*, MAPKs, c-Cbl, antifungal immunity

## Abstract

Non-receptor tyrosine kinase c-Abl is critical for host defense against bacterial and viral infections, yet its role in antifungal immunity remains elusive. Here, we report that inhibition of c-Abl with flumatinib mesylate significantly impairs the survival rate and exacerbates fungal burden in mice infected with *Candida albicans*. Our findings reveal that c-Abl inhibition reduces production of TNF-α, IL-10, and IL-12 in bone marrow-derived dendritic cells (BMDCs) after stimulation with fungal β-glucan or α-mannan. Mechanistically, c-Abl inhibition significantly blocks p38 and extracellular signal-regulated kinases 1/2 (ERK1/2) activation in BMDCs after α-mannan stimulation in a c-Cbl dependent manner. Collectively, our study uncovers a c-Abl/c-Cbl/MAPK signaling axis in dendritic cells that governs antifungal innate immunity, highlighting c-Cbl as a critical downstream mediator linking c-Abl to host defense against *C. albicans*. Our findings provide a mechanistic basis for fungal risk assessment in cancer patients treated with c-Abl inhibitors.

## INTRODUCTION

Invasive fungal infection represents a growing clinical challenge, accounting for millions of deaths annually (1). *Candida albicans*, an opportunistic fungal pathogen, is the predominant cause of fungal infection in immunocompromised hosts (2). Current antifungal therapies, including azoles and echinocandins, are limited by rising resistance rates and host toxicity (3). During fungal invasion, innate immune cells detect pathogen-associated molecular patterns (PAMPs) via C-type lectin receptors (CLRs), initiating spleen tyrosine kinase (Syk) phosphorylation and activation. The activated Syk promotes caspase recruitment domain family member 9 (CARD9) recruitment and activation, triggering pro-inflammatory cytokine release (4). Elucidating the molecular mechanisms underlying antifungal immunity is critical for the development of immune-modulatory therapeutic strategies.

c-Abl is a non-receptor protein tyrosine kinase, which is ubiquitously expressed in almost all tissues and cell types. It has been found fused to a variety of translocation partners in leukemias, the oncogenic activity of which drives the development of certain human leukemias. Accumulating evidence has demonstrated that c-Abl can be activated by a variety of intracellular and extracellular stress signals and is involved in regulation of cell proliferation, cytoskeleton remodeling, cell adhesion, and DNA damage repair (5–7). Recently, c-Abl has been reported to regulate the process of bacterial and viral infection (8, 9), and its specific inhibitor, flumatinib mesylate, is widely used for cancer patients. Several studies have found that fungal infections are frequently observed in patients treated with c-Abl inhibitors, suggesting that c-Abl may protect those patients from fungal infection (10–12). Whether c-Abl modulates antifungal responses via CLRs remains unknown.

In this study, we reveal that during systemic *C. albicans* infection, c-Abl functions as a critical signal transducer, bridging CLR engagement to MAPK activation through the E3 ubiquitin ligase c-Cbl. These findings not only uncover a previously uncharacterized CLR-c-Abl/c-Cbl signaling axis in dendritic cells but also provide a mechanistic basis for clinical fungal risk assessment in tumor patients receiving c-Abl inhibitor therapy. By defining c-Abl as a pivotal mediator of antifungal innate immunity, our study highlights the need for integrated monitoring of fungal susceptibility in c-Abl-targeted cancer therapies.

## RESULTS

### CLR mediates *C. albicans*-induced c-Abl activation

**Fig 1:**
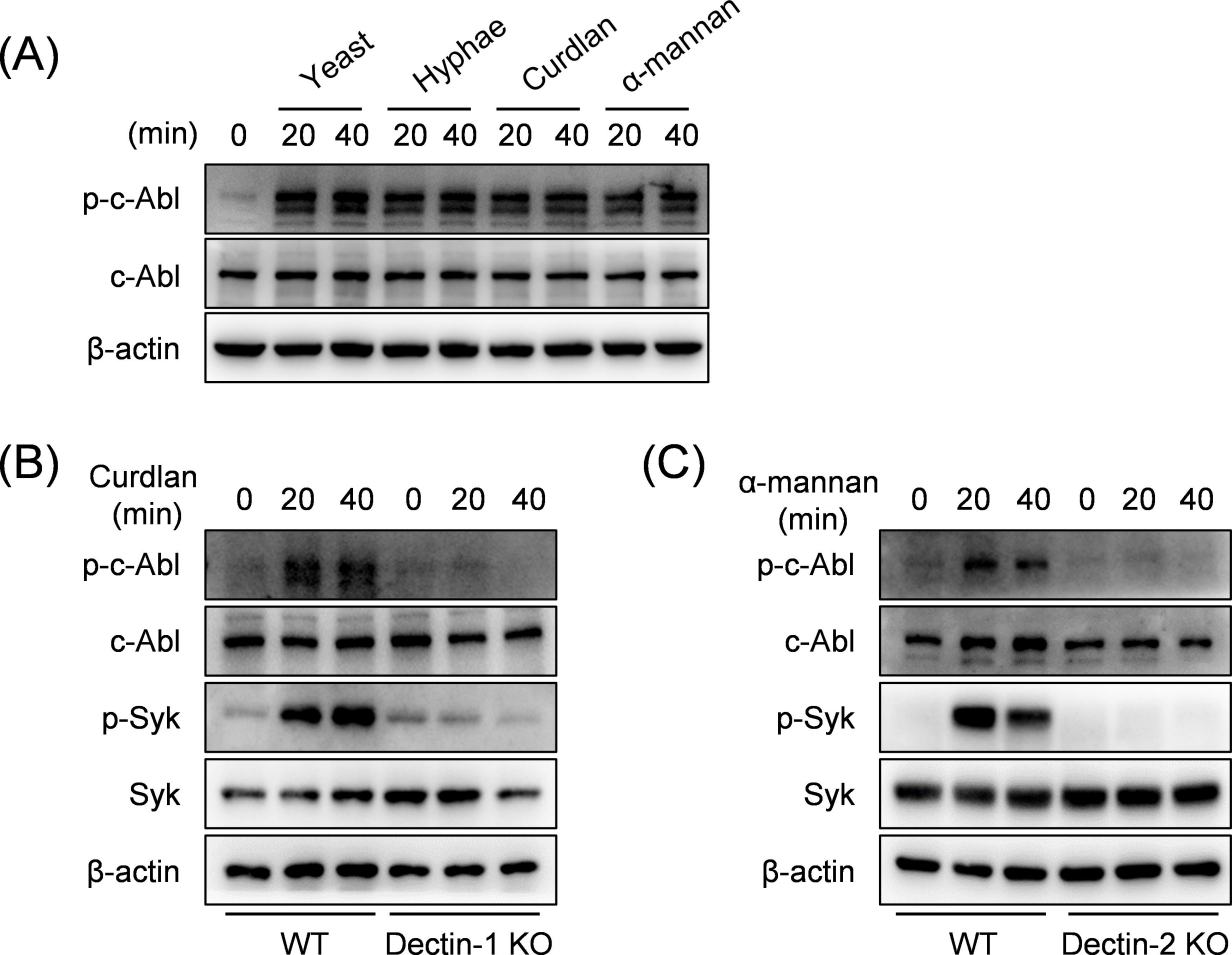
CLR mediates *C. albicans*-induced c-Abl activation. (**A**) Western blot analysis of the indicated proteins in BMDCs stimulated with yeast, hyphae, Curdlan, and α-mannan. (**B and C**) Western blot analysis of the indicated proteins in BMDCs derived from WT and Dectin-1 KO mice stimulated with Curdlan (**B**) or Dectin-2 KO mice stimulated with α-mannan (**C**).

The involvement of c-Abl in anti-viral and anti-bacterial immunity has been extensively documented (13). However, its role in antifungal immune responses remains poorly characterized. We discovered that stimulation of wild-type (WT) BMDCs with *C. albicans* yeast, hyphae, or fungal cell wall polysaccharides β-glucan containing Curdlan and α-mannan induced a significant increase in phosphorylation of c-Abl (Fig. 1A). Consistently, Curdlan or α-mannan induced phosphorylation of c-Abl was abrogated in cells deficient in CLRs, including Dectin-1 and Dectin-2, which have been confirmed to recognize Curdlan and α-mannan (14, 15). Phosphorylation of Syk, a pivotal kinase coupled to CLRs, was similarly abolished in KO cells, indicating a coordinated signaling cascade (Fig. 1B and C). These findings indicate that c-Abl is involved in Syk-coupled CLR signaling pathways and is phosphorylated in response to *C. albicans* infection.

### Pharmacological inhibition of c-Abl enhances susceptibility to systemic *C. albicans* infection in mice

**Fig 2:**
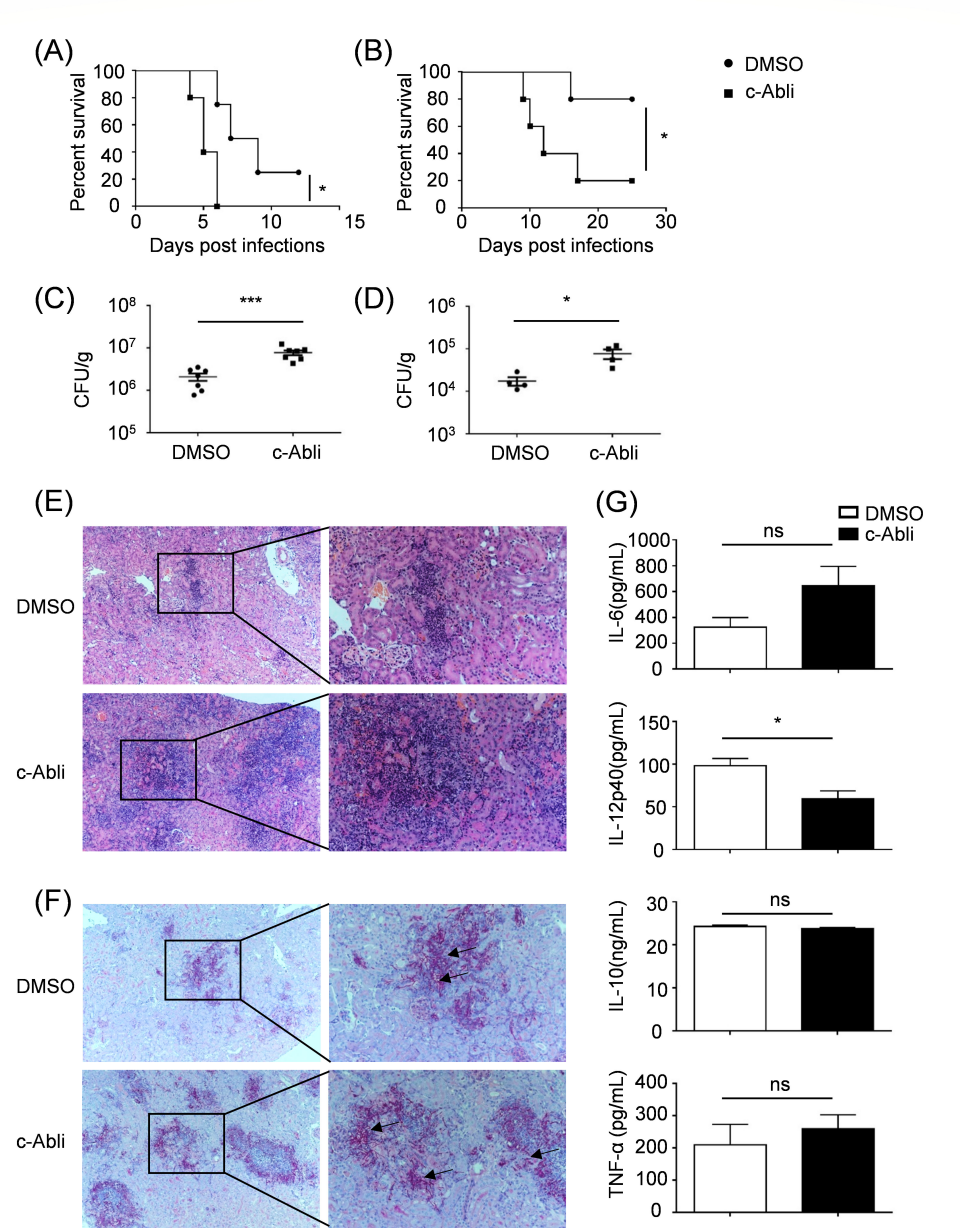
c-Abl inhibition increases susceptibility to systemic *C. albicans* infection in mice. (**A and B**) Survival rates of mice treated with DMSO or c-Abl inhibitor post 1.5 × 10^5^ (**A**) or 5 × 10^4^ (**B**) *C. albicans* infection. (**C and D**) Fungal burden in kidneys of mice treated with DMSO or c-Abl inhibitor 2 days post 1.5 × 10^5^ (**C**) or 5 × 10^4^ (**D**) *C. albicans* infection. (**E and F**) H&E (**E**) and PAS (**F**) staining of kidneys from 1.5 × 10^5^ *C. albicans-*infected mice treated with DMSO or c-Abl inhibitor. (**G**) ELISA analysis of indicated cytokines in renal homogenates from mice treated with DMSO or c-Abl inhibitor post *C. albicans* infection. ^ns^*P* > 0.05, *P < 0.05, ***P < 0.001. *P* < 0.05 indicated a statistically significant difference between experimental groups.

To delineate the functional role of c-Abl in antifungal immunity *in vivo*, we administered the c-Abl-specific inhibitor flumatinib mesylate to suppress the kinase activity of c-Abl. Pharmacological inhibition of c-Abl significantly reduced the survival rates of mice compared with the DMSO vehicle control group during both low- and high-dose *C. albicans* infections (Fig. 2A and B). Concurrent analysis of fungal dissemination revealed elevated fungal burdens in kidneys and livers of inhibitor-treated mice relative to controls (Fig. 2C and D). Histopathological examination demonstrated exacerbated renal pathology in c-Abl-inhibited mice, characterized by increased inflammatory foci density and inflammatory infiltration (Fig. 2E). Periodic acid-Schiff (PAS) staining further identified enhanced fungal invasion patterns in treated kidneys, exhibiting greater hyphal density and tissue penetration compared with controls (Fig. 2F). To mechanistically link these phenotypes to immune modulation, we quantified cytokine levels in renal homogenates post-infection. c-Abl inhibition selectively attenuated IL-12p40 production, while IL-6, IL-10, and TNF-α levels remained comparable to the DMSO group (Fig. 2G). These findings indicated that c-Abl plays a protective role in host antifungal immunity.

### c-Abl mediates *C. albicans*-induced pro-inflammatory cytokine production

**Fig 3:**
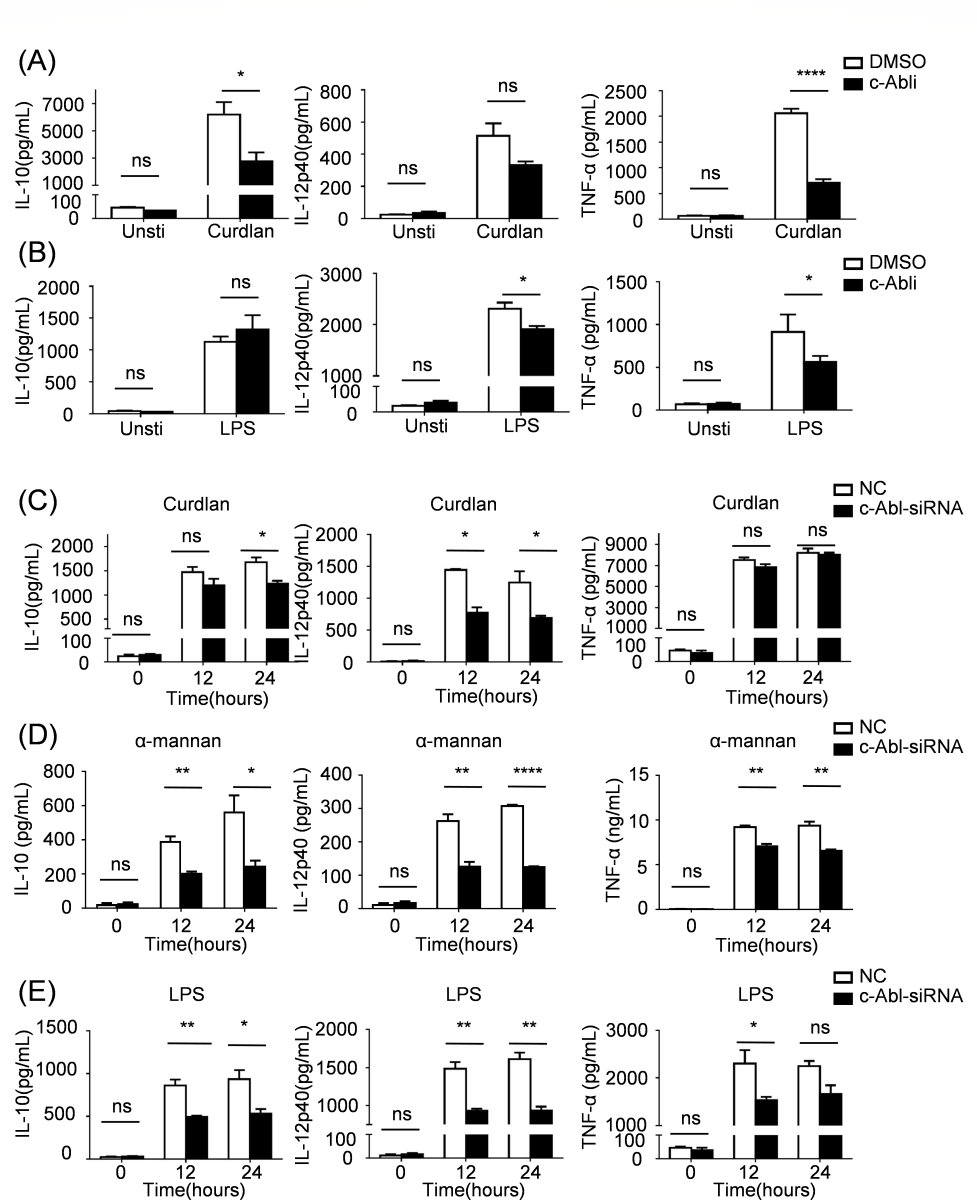
c-Abl mediates *C. albicans*-induced pro-inflammatory cytokine production. ELISA analysis of IL-10, IL-12p40, and TNF-α in supernatants from BMDCs pre-treated with DMSO or c-Abl inhibitor after stimulation with Curdlan (**A**) and LPS (**B**) for 24 h. ELISA analysis of IL-10, IL-12p40, and TNF-α in supernatants from BMDCs pre-treated with NC-siRNA or c-Abl-siRNA after stimulation with Curdlan (**C**), α-mannan (**D**), or LPS (**E**) at indicated times. ^ns^*P* > 0.05, *P < 0.05, **P < 0.01, ***P < 0.001, ****P < 0.0001. *P* < 0.05 indicated a statistically significant difference between experimental groups.

Next, we determined the effect of c-Abl on cytokine production in BMDCs. Consistent with previous reports (16), both pharmacological inhibition and genetic knockdown (siRNA) of c-Abl significantly attenuated IL-10, IL-12p40, and TNF-α secretion in LPS-stimulated BMDCs relative to controls (Fig. 3B and E). Intriguingly, we also found that c-Abl inhibitor or c-Abl-specific siRNA treatment decreased IL-10, IL-12p40, and TNF-α production in BMDCs after Curdlan (Fig. 3A and C) and α-mannan (Fig. 3D) stimulation compared with that in the control group. These coordinated findings demonstrate that c-Abl operates downstream of CLRs to potentiate their pro-inflammatory cytokine induction in response to fungal infection.

### c-Abl is not involved in fungal phagocytosis

**Fig 4:**
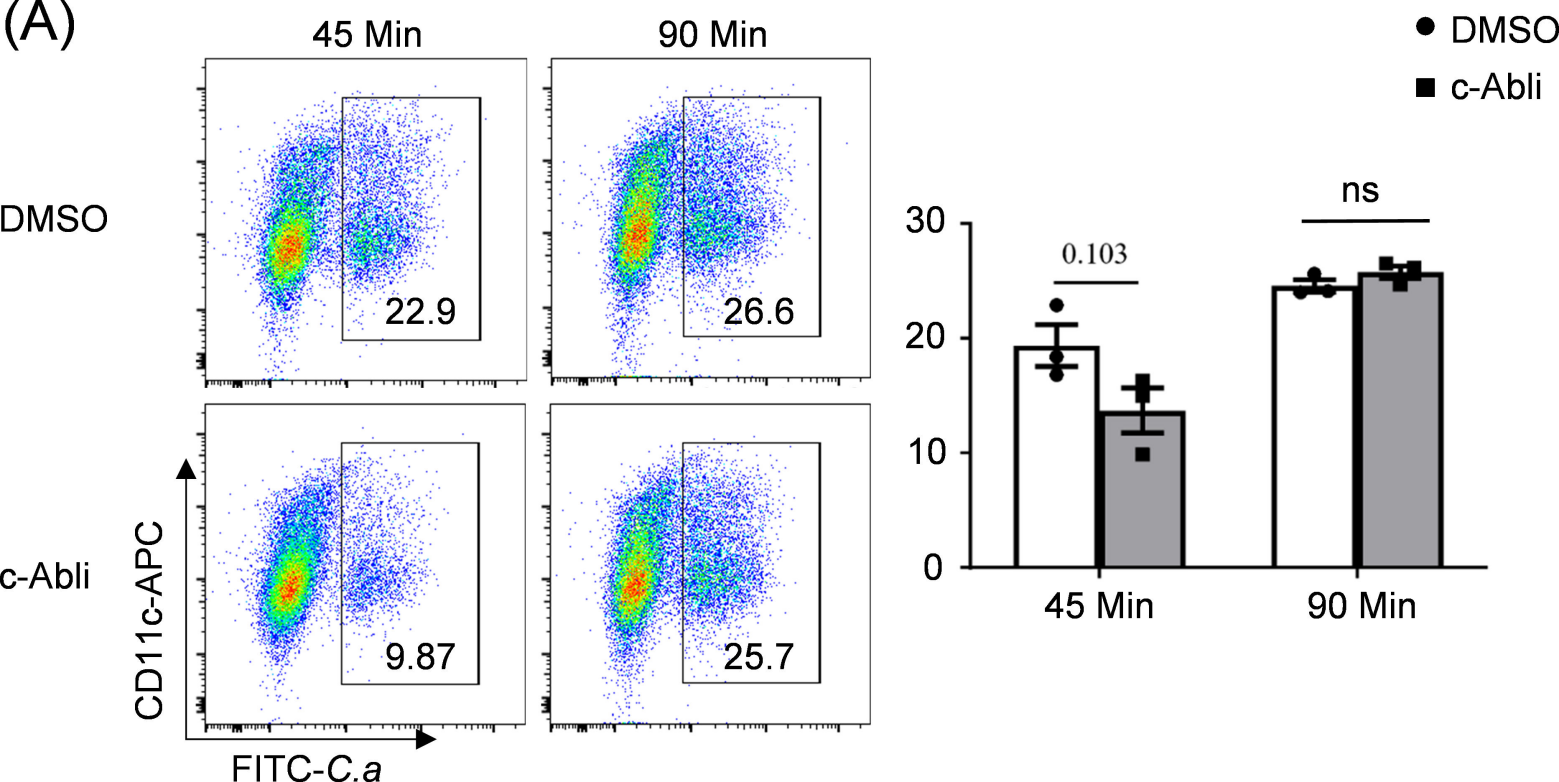
c-Abl is not involved in fungal phagocytosis. (**A**) Flow cytometry analysis of the phagocytosis of GFP-labeled *C. albicans* and phagocytic frequencies in DC2.4 pre-treated with DMSO or c-Abl inhibitor for 45 or 90 min. ^ns^*P* > 0.05. *P* < 0.05 indicated a statistically significant difference between experimental groups.

c-Abl has been demonstrated to participate in cytoskeletal reorganization (17), while dendritic cells serve as professional phagocytes that orchestrate antifungal immunity through pathogen engulfment and antigen presentation (18, 19). We sought to investigate whether c-Abl exerts its functional effects by modulating the phagocytic activity of BMDCs against *C. albicans*. To investigate whether c-Abl regulates this critical function, we quantified phagocytic capacity using FITC-labeled *C. albicans* in BMDCs. Pharmacological inhibition of c-Abl demonstrated no significant impairment in fungal uptake by DC2.4 cells (a murine DC line) compared with vehicle-treated controls, as assessed by flow cytometry over 45- and 90-min incubation periods (Fig. 4A).

### c-Abl regulates the p38 and ERK MAPK pathways in a c-Cbl-dependent manner

**Fig 5:**
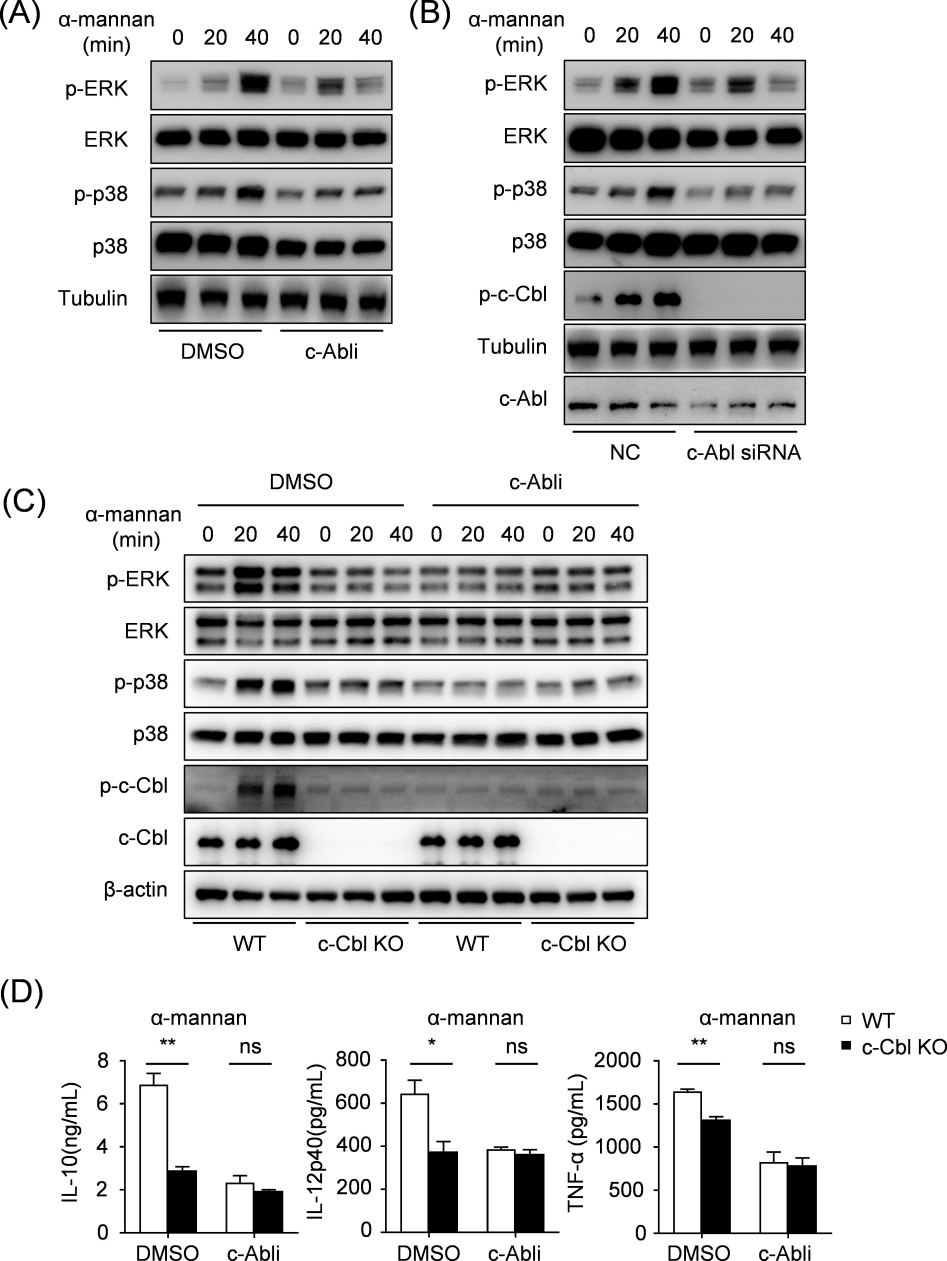
c-Abl regulates the p38 and ERK MAPK pathway in a c-Cbl dependent manner. (**A**) Western blot analysis of the indicated proteins in BMDCs pre-treated with DMSO or c-Abl inhibitor after stimulation with α-mannan. (**B**) Western blot analysis of the indicated proteins in BMDCs pre-treated with NC-siRNA or c-Abl-siRNA after stimulation with α-mannan. (**C**) Western blot analysis of the indicated proteins in WT or c-Cbl KO BMDCs pre-treated with DMSO or c-Abl inhibitor after stimulation with α-mannan. (**D**) ELISA analysis of IL-10, IL-12p40, and TNF-α in supernatants from WT and c-Cbl KO BMDCs pre-treated with DMSO or c-Abl inhibitor after stimulation with α-mannan. ^ns^*P* > 0.05, *P < 0.05, **P < 0.01. *P* < 0.05 indicated a statistically significant difference between experimental groups.

Previous studies have shown that c-Abl plays an important role in regulating the p38 and ERK signaling pathways (20, 21). The Dectin-1-CARD9 signaling pathway can also exert antifungal immunity by activating ERK (22). To explore the intracellular signaling pathway of c-Abl in BMDCs after *C. albicans* stimulation, we detected the phosphorylation of p38 and ERK MAPK signaling pathway by Western blot. The results showed that α-mannan stimulation significantly induced phosphorylation of p38 and ERK, and it could be eliminated by c-Abl inhibitor (Fig. 5A) or siRNA-mediated gene knockdown (Fig. 5B). These data indicate that c-Abl mediates the activation of p38 and ERK signaling pathways in response to fungal infection.

Casitas B-lineage lymphoma (c-Cbl) is known as an E3 ubiquitin ligase, which plays an important role in host antifungal immunity (23). Our previous studies have demonstrated that in DSS-induced experimental colitis, c-Abl mediates the phosphorylation of c-Cbl, thereby promoting IL-10 production (24). In this study, our observation reveals that deficiency of c-Abl resulted in the undetectable phosphorylation of c-Cbl (Fig. 5B). Combining our findings with prior reports, the activation of c-Abl is intrinsically linked to c-Cbl phosphorylation activation.

To investigate whether c-Cbl is involved in the c-Abl-activated MAPK signaling pathway, we measured the phosphorylation of ERK, p38, and c-Cbl in WT and c-Cbl-deficient (c-Cbl KO) BMDCs pre-treated with a c-Abl inhibitor or DMSO control, followed by α-mannan stimulation. We found that the absence of c-Cbl alone failed to induce ERK/p38 phosphorylation, and inhibition of c-Abl significantly abolished ERK/p38 activation in both WT and c-Cbl knockout (KO) cells. (Fig. 5C). Meanwhile, we analyzed cytokine levels of IL-10, IL-12p40, and TNF-α in WT and c-Cbl KO BMDCs pre-treated with c-Abl inhibitor and DMSO control after α-mannan stimulation. We found that inhibition of c-Abl eliminated the difference of the pro-inflammatory cytokines production between WT and c-Cbl KO BMDCs (Fig. 5D). Considered together, c-Abl regulates the p38 and ERK MAPK pathways in a c-Cbl-dependent manner. These data indicate that c-Abl and c-Cbl jointly mediate the phosphorylation and activation of p38 and ERK during *C. albicans* infection.

### c-Abl/c-Cbl axis regulates innate antifungal immunity

**Fig 6:**
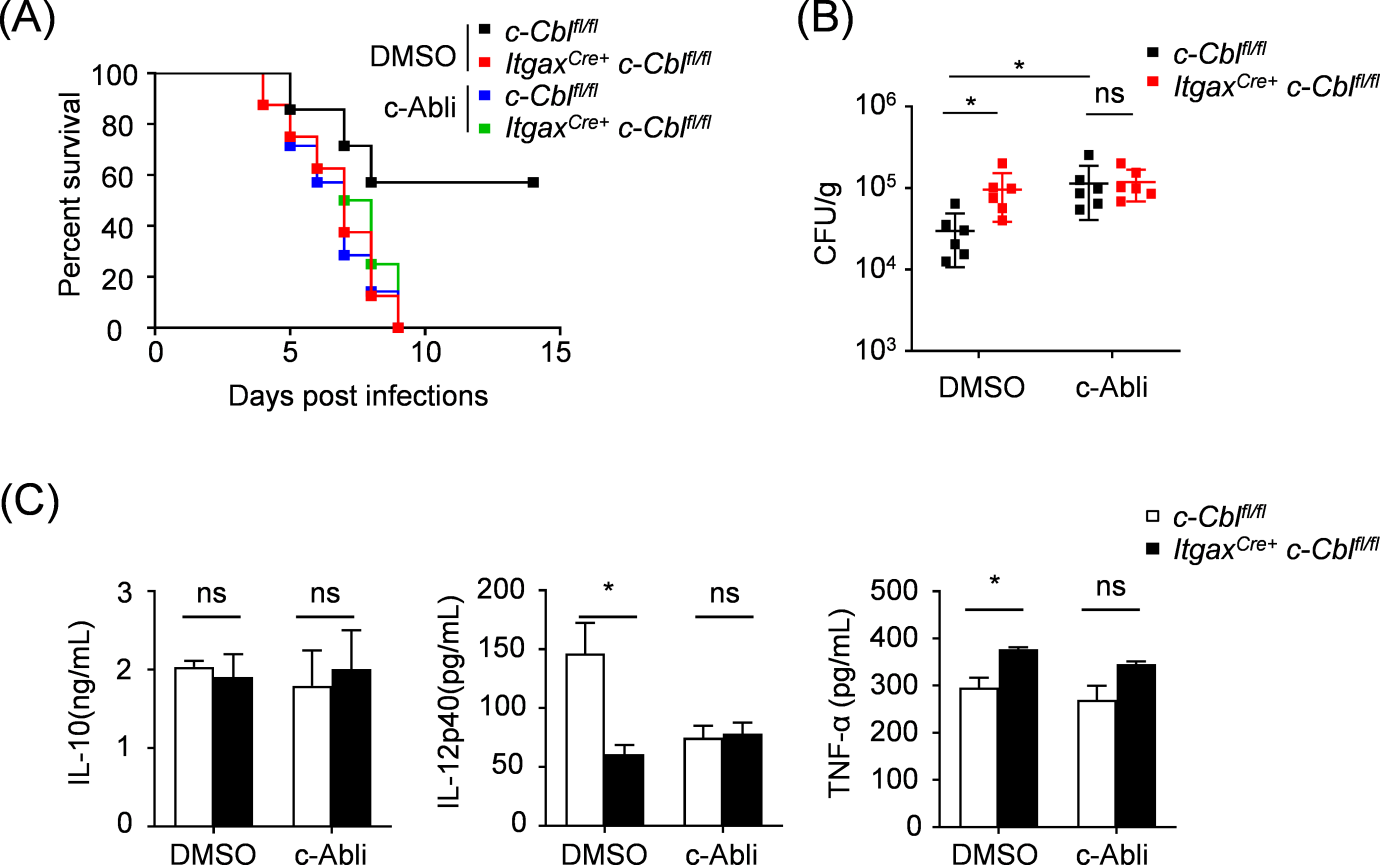
c-Abl/c-Cbl axis regulates innate antifungal immunity. (**A**) Survival rates of *c-Cbl^fl/fl^* and *Itgax^Cre+^ c-Cbl^fl/fl^* mice treated with DMSO or c-Abl inhibitor post *C. albicans* (1.5 × 10^5^) infection. (**B**) Fungal burden in kidneys from *c-Cbl^fl/fl^* and *Itgax^Cre+^ c-Cbl^fl/fl^* mice treated with DMSO or c-Abl inhibitor 2 days post *C. albicans* (1.5 × 10^5^) infection. (**C**) ELISA analysis of IL-10, IL-12p40, and TNF-α in renal homogenate supernatants from *c-Cbl^fl/fl^* and *Itgax^Cre+^ c-Cbl^fl/fl^* mice treated with DMSO or c-Abl inhibitor 2 days post *C. albicans* (1.5 × 10^5^) infection. ^ns^*P* > 0.05, *P < 0.05. *P* < 0.05 indicated a statistically significant difference between experimental groups.

Our previous work has proved that in macrophages, c-Cbl is involved in host anti-*C*. *albicans* infections (25). To explore how the c-Abl/c-Cbl axis works in host antifungal immunity, we utilized a systemic *C. albicans* infection model involving dendritic cell-specific c-Cbl-deficient mice (*Itgax^Cre/+^ c-Cbl^fl/fl^*) and their *c-Cbl^fl/fl^* control littermates, both treated with a c-Abl inhibitor or DMSO. Our experiments revealed a much higher susceptibility to *C. albicans* in *Itgax^Cre/+^ c-Cbl^fl/fl^* mice than *c-Cbl^fl/fl^* mice, and this difference was eliminated by c-Abl inhibitor treatment (Fig. 6A and B). Besides, the production of pro-inflammatory cytokines IL-12p40 and TNF-α showed significant differences between *Itgax^Cre/+^ c-Cbl^fl/fl^* and *c-Cbl^fl/fl^* mice, whereas c-Abl inhibitor resolved the aforementioned discrepancies (Fig. 6C). Collectively, these findings delineate a previously uncharacterized role for the CLR-c-Abl/c-Cbl signaling axis in orchestrating antifungal innate immunity.

## DISCUSSION

Initially identified for its role in cancer development, particularly in chronic myeloid leukemia and other hematologic malignancies, c-Abl is a non-receptor tyrosine kinase (5). Multiple specific small-molecule inhibitors targeting c-Abl or BCR-ABL have been developed, demonstrating their significant therapeutic efficacy in the clinical management of leukemia and other malignancies (26). Recent clinical case studies have reported an association between c-Abl-targeted small-molecule inhibitors and increased susceptibility to fungal infections. Specifically, mucormycosis has been documented in patients with Philadelphia chromosome-positive acute lymphoblastic leukemia treated with imatinib, a c-Abl inhibitor (11). Moreover, patients receiving dasatinib therapy exhibit a heightened risk of fungal infections (12). In our study, by the application of the specific c-Abl inhibitor flumatinib mesylate, we demonstrate that c-Abl plays a critical role in host antifungal immunity. c-Abl inhibition increases the susceptibility to invasive fungal infection in mice, with important implications for clinical leukemia therapy.

CLR family members Dectin-1 and Dectin-2, which recognize fungal cell wall polysaccharides β-glucan and α-mannan, respectively, initiate immune responses and serve as major receptors in host antifungal immunity. Here, we show that *C. albicans*-induced c-Abl phosphorylation is dependent on Syk-coupled CLR signaling, expanding the known role of CLR pathways in antifungal immunity. Conventionally, upon fungal pathogen recognition, CLRs recruit and activate spleen tyrosine kinase Syk, inducing phosphorylation and activation of CARD9. This promotes formation of a ternary complex with BCL-10 and MALT-1 to regulate MAPK and NF-κB signaling, ultimately modulating production of pro-inflammatory cytokines (TNF-α, IL-6, IL-1β) and reactive oxygen species to initiate antifungal immunity (27, 28). Notably, CARD9 exhibits its pathway plasticity through its interaction with H-Ras and Ras-GRF1, selectively amplifying ERK1/2 activation to modulate cytokine production (22). In this case, we investigated the relationship between c-Abl and the MAPK signaling pathway, which is likewise co-regulated by CLRs, and its role in mediating fungal infection-induced inflammatory cytokine production. Our findings reveal a novel mode of c-Abl-mediated MAPK signaling regulation during fungal infection.

The E3 ubiquitin ligase c-Cbl is known to act as a negative regulator in lymphocytes by mediating ubiquitination and degradation of receptors and kinases (23, 29, 30). Our previous study identified c-Cbl as a key regulator in maintaining intestinal homeostasis through suppressing fungi-induced noncanonical NF-κB activation. Mice with dendritic cell-specific deficiency of c-Cbl exhibit aggravated colitis because P65-mediated *Il-10* transcription is inhibited by mannan-induced activation of RelB (24). Here, we show that c-Abl controls fungal-induced c-Cbl activation and specifically regulates MAPK signaling. Conditional deletion of c-Cbl in dendritic cells abrogates the effect of c-Abl inhibition on host antifungal immunity, demonstrating that c-Abl regulates p38 and ERK MAPK activation via c-Cbl to promote antifungal immune responses.

Collectively, our study identifies c-Abl as a key regulator of antifungal innate immunity, offering a mechanistic explanation for the increased susceptibility to fungal infections observed in patients treated with c-Abl inhibitors. These results highlight the need for clinical monitoring of fungal risk in c-Abl-targeted therapy.

## MATERIALS AND METHODS

### Mice

All mice (C57BL/6) were housed at Tongji University under specific pathogen-free conditions. Dendritic cell-specific c-Cbl-deficient mice (*Itgax^Cre+^ c-Cbl^fl/fl^*) and their littermate control (*c-Cbl^fl/fl^*) were described as before (24).

### Fungal strain and culture condition

*C*. *albicans* (SC5314) colonies were grown from frozen stocks by streaking on YPD (yeast extract, peptone, and dextrose) agar plates. Yeast cells were cultured in YPD-rich medium at 30°C for 16 h. The cells were then pelleted (6,000 × *g*, 1 min), washed, and resuspended in PBS buffer for subsequent experiments.

### Mouse model of systemic candidiasis

Mice were infected with 1.5 × 10^5^ or 5 × 10^4^ CFU of *C. albicans* through the lateral tail vein and monitored daily for health and survival. For fungal burden determination, homogenized kidneys were diluted with PBS and plated on SDA agar. The number of CFUs was counted after incubation at 30°C for 48 h, and the fungal loads were calculated according to the organ weight.

### Antibodies and reagents

The antibodies and reagents used in this work are listed in Tables 1 and 2.

**TABLE 1:** Antibodies used for immunoblot

Antibodies	Catalog number	Supplier
phospho-ERK	9101	Cell Signaling Technology
phospho-p38	4511	Cell Signaling Technology
p38	8690	Cell Signaling Technology
c-Abl	2862	Cell Signaling Technology
phospho-c-Cbl	8869	Cell Signaling Technology
c-Cbl	2747	Cell Signaling Technology
phospho-Syk	2710	Cell Signaling Technology
Syk	13198	Cell Signaling Technology
β-Tubulin	2148	Cell Signaling Technology
β-Actin	4970	Cell Signaling Technology
ERK	sc-154	Santa Cruz Biotechnology
Phospho-c-Abl	110403	Sigma-Aldrich
Goat anti-mouse IgG HRP	M21001	Abmart
Goat anti-rabbit IgG-HRP	M21002	Abmart

**TABLE 2:** Reagents used in this work

Reagents	Catalog number	Supplier
RIPA Lysis buffer	P0013C	Beyotime Biotechnology
Phenylmethanesulfonyl fluoride	ST505	Beyotime Biotechnology
Phosphatase inhibitor cocktail	P1045	Beyotime Biotechnology
flumatinib mesylate	HY-13905	MedChemExpress
GM-CSF	RMGMCSF20	PeproTech
LPS	L5024	Sigma-Aldrich
Curdlan	C7821	Sigma-Aldrich
α-Mannan	M7054	Sigma-Aldrich
Trypan blue	72-57-1	Sigma-Aldrich

### Phagocytosis of *C. albicans*

Phagocytosis of *C. albicans* by DC2.4 cells was assessed by flow cytometry based on previously reported methods (31). Briefly, *C. albicans* yeast cells (1 × 10^8^/mL) were suspended in 1 μg/mL fluorescein isothiocyanate (FITC) in 0.05 M carbonate-bicarbonate buffer (pH 9.5) for 15 min at room temperature in the dark. After incubation, FITC-labeled *C. albicans* yeast cells were washed twice with PBS. FITC-*C. albicans* (1 × 10^7^/mL) was incubated with DC2.4 cells (1 × 10^6^) at 37°C for the indicated time. After washing three times with ice-cold PBS, the DC2.4 cells were collected. The cells were re-suspended in Trypan blue (200 μg/mL) solution and incubated for 10 min to quench fluorescence of non-internalized FITC-*C. albicans*. After washing the cells with staining buffer, anti-mouse CD11c-APC (BioLegend, USA) was added and stained for 30 min at 4°C. Data were analyzed with FlowJo software.

### Preparation of bone marrow-derived dendritic cells (BMDCs)

BMDCs were generated by cultivating bone marrow cells in RPMI 1640 medium containing 10% FBS and 100 U/mL penicillin and streptomycin supplemented with recombinant murine GM-CSF (20 ng/mL). Fresh GM-CSF-containing medium was added on days 3 and 6, and BMDCs were collected at day 8 for subsequent experiments. The generated dendritic cell population was analyzed by flow cytometry based on CD11c expression.

### Western blot

BMDCs were serum-starved overnight and then stimulated. Cells were collected using RIPA lysis buffer pre-added with protease inhibitor (PMSF) and phosphatase inhibitor cocktail. Proteins were separated by 10% SDS-PAGE and transferred to PVDF membranes. Primary antibodies used in this study are shown in Table 1.

### siRNA-mediated gene knockdown

Lipofectamine 3000 transfection reagent (Thermo Fisher Scientific, USA) was used to introduce siRNA into BMDCs. For siRNA transfection, the siRNA targeting mouse *c-Abl* and the non-targeting control siRNA (NC) or Lipofectamine 3000 were individually mixed with 100 μL Opti-MEM (Gibco) for 5 min. Subsequently, the siRNA/Lipofectamine 3000-containing Opti-MEM solutions were combined for 15 min and added to the cell culture medium. The efficiency of knockdown was evaluated by Western blot.

### Cytokine measurement

The supernatants of homogenized kidneys from mice infected with *C. albicans* were collected. BMDCs were stimulated with α-mannan, Curdlan, or LPS for 12 or 24 h. Supernatants of BMDCs were collected. The protein levels of TNF-α, IL-6, IL-10, and IL-12p40 were determined by immunosorbent assay kits (eBioscience, USA) according to the manufacturer’s protocol.

### Histopathology

The kidneys were collected from mice treated with or without c-Abl inhibitor after *C. albicans* infection. Kidneys were fixed with 4% formaldehyde solution. H&E staining and PAS staining were performed for histopathological analysis.

### Statistical analysis

Data were integrated from three independent experiments. Data are presented as mean ± SEM, and comparisons between two groups were performed using the unpaired two-tailed Student’s t-test. Gehan-Breslow-Wilcoxon test was performed for survival analysis of mice. Data were analyzed using GraphPad Prism 8. *P* < 0.05 was considered statistically significant.
